# Altered Expression of Genes in Signaling Pathways Regulating Proliferation of Hematopoietic Stem and Progenitor Cells in Mice with Subchronic Benzene Exposure

**DOI:** 10.3390/ijerph120809298

**Published:** 2015-08-07

**Authors:** Rongli Sun, Juan Zhang, Mengzhen Xiong, Haiyan Wei, Kehong Tan, Lihong Yin, Yuepu Pu

**Affiliations:** Key Laboratory of Environmental Medicine Engineering, Ministry of Education, School of Public Health, Southeast University, No.87 Dingjia Qiao, Gulou District, Nanjing 210009, China; E-Mails: sunrongli20609@163.com (R.S.); xmzseu@hotmail.com (M.X.); why_314614@163.com (H.W.); kehom123@126.com (K.T.); lhyin@seu.edu.cn (L.Y.)

**Keywords:** benzene, post-exposure toxicity, hematopoietic stem cell, Wnt, Notch, Hedghog

## Abstract

Leukemias and hematopoietic disorders induced by benzene may arise from the toxicity of benzene to hematopoietic stem or progenitor cells (HS/PCs). Since there is a latency period between initial benzene exposure and the development of leukemia, subsequent impact of benzene on HS/PCs are crucial for a deeper understanding of the carcinogenicity and hematotoxicity in post-exposure stage. This study aims to explore the effects of benzene on HS/PCs and gene-expression in Wnt, Notch and Hh signaling pathways in post-exposure stage. The C3H/He mice were injected subcutaneously with benzene (0, 150, 300 mg/kg/day) for three months and were monitored for another 10 months post-exposure. The body weights were monitored, the relative organ weights, blood parameters and bone marrow smears were examined. Frequency of lineage^-^ sca-1^+^ c-kit^+^ (LSK) cells, capability of colony forming and expression of genes in Wnt, Notch and Hedghog (Hh) signaling pathways were also analyzed. The colony formation of the progenitor cells for BFU-E, CFU-GEMM and CFU-GM was significantly decreased with increasing benzene exposure relative to controls, while no significant difference was observed in colonies for CFU-G and CFU-M. The mRNA level of cyclin D1 was increased and Notch1 and p53 were decreased in LSK cells in mice exposed to benzene but with no statistical significance. These results suggest that subsequent toxic effects of benzene on LSK cells and gene expression in Wnt, Notch and Hh signaling pathways persist in post-exposure stage and may play roles in benzene-induced hematotoxicity.

## 1. Introduction

Benzene is a common solvent and widely used in various industries. It is also an environmental pollutant present in cigarette smoke and vehicle exhaust [1]. Benzene is classified as a Group 1 Carcinogen by The International Agency for Research on Cancer (IARC) [2]. The hematopoietic system is the chief target of benzene’s toxic effects which manifest as alterations in the levels of circulating blood cells. Increasing risk of acute myeloid leukemia (AML), myelodysplastic syndrome and other hematological malignancies can be found in benzene-exposed workers [3,4,5].

The bone marrow is a critical target organ for benzene metabolites, and both HS/PCs and stromal cells are potential targets of hematotoxicity induced by benzene. Exposure to benzene induces gene mutations [6], oxidative stress [7] and inappropriate gene expression [8]. These alterations may abnormally activate self-renewal and differentiation signaling pathways in HS/PCs, thus resulting in aberrant expression of downstream genes and malignant transformation and dysfunction of HS/PCs.

Although a large number of studies on potential health hazards associated with benzene exposure have been done, information is limited on hematopoietic function and alterations of genes related to self-renewal and differentiation signaling pathways of HS/PCs in benzene exposed mice long after the period of initial exposure. Since the onset of effects of prolonged benzene exposure may be delayed for months or years after the actual exposure has ceased, studying these gene expression changes may give us a clue for evaluation of the risk of leukemia and prevention of progression of hematopoietic malignancy.

The Wnt, Notch and Hh signaling pathways are pivotal regulatory pathways related to self-renewal and differentiation. Irregularities in these three signaling pathways are highly associated with hematopoietic malignancies [9]. The Wnt signaling pathway is required for normal HSC growth and plays an important role in self-renewal and proliferation of HS/PCs, suggesting an involvement in leukemogenesis [10]. The important mediator of the canonical Wnt pathway is β-catenin that is expressed at various protein levels in AML patients [11]. As assessed in vitro, β-catenin expression was correlated with self-renewal of leukemic cells [12]. Dysregulated Wnt signaling has been identified as a key factor in the initiation of various kinds of cancer. Two Wnt/β-catenin downstream target genes, c-myc and cyclin D1, are known to be involved in the oncogenic function of inappropriate activation of Wnt signaling [13,14].

Notch signaling pathway plays a fundamental role in regulating hematopoietic development and genes in Notch pathway function as oncogenes or tumor suppressors in several cancers [15,16]. There is an interaction between Notch and Wnt signaling pathway to maintain HSCs in an undifferentiated state. Duncan *et al.* demonstrated that Notch1 was upregulated in response to Wnt signaling in HSCs and Wnt may exert its influence by activating Notch1 targets genes [17]. Lobry *et al.* found that the Notch1 mRNA level was significantly less in AML patient samples and mouse model in comparison with normal HSPCs [18]. Cyclin D1 is a downstream target gene in the Notch pathway. There is up-regulation of cyclin D1 gene expression in MDS patients with monosomy 7 and trisomy 8, and in those with trisomy 8 AML [19,20].

The Hh signaling pathway has been closely associated with cell cycle regulation and is critical for self-renewal and proliferation of stem cells [21,22]. In the adult, Hh signaling is involved in tissue maintenance and repair and regulation stem cell behavior. Aberrant activation of Hh signaling is implicated in multiple aspects of transformation, including the maintenance of cancer stem cell (CSC) phenotype [23]. Bmi 1 has shown to be induced by Hh signaling and is important for stem cell self-renewal [22]. p53, a tumor suppressor protein, is one of the downstream effectors of Bmi 1. In HSCs, p53 is preferentially expressed in LSK cells where it negatively regulates self-renewal and maintains quiescence [24]. Park *et al.* demonstrated that loss of Bmi 1 in HSCs resulted in impairment of HSC self-renewal owing to accumulation of p19^Arf^, which causes p53-dependent apoptosis [25].

In this study, we exposed mice to benzene for 3 months, stopped benzene exposure for 10 months and then investigated the frequency and colony formingability of LSK cells in benzene-exposed mice. Key and targeted genes of Wnt, Notch and Hh signaling pathways, which are related with self-renewal and differentiation, were also evaluated by nest real-time PCR analysis. The cyclin D1 mRNA level was elevated, while the Notch1 and p53 were declined in LSK cells in mice following 10 months post-benzene exposure. These results suggest that cyclin D1, Notch1 and p53 may be the genes affected by benzene exposure and may play roles in the development of benzene-induced hematotoxicity.

## 2. Materials and Methods

### 2.1. Chemicals

Benzene was purchased from Sigma-Aldrich (St. Louis, MO, USA). Corn oil was purchased from COFCO (Beijing, China).

### 2.2. Animals and Treatments

Male C3H/He mice were (aged 4 weeks) obtained from Wei Tong Li Hua Laboratory Animal Co. Ltd. (Beijing, China). Animals were maintained under a 12-h light/12-h dark cycle at a temperature of 25 ± 2 °C with a relative humidity of 45%–65%. After one week of acclimatization, the mice were divided into 3 groups (*n* = 20/group): Control group (C: vehicle, oil); Benzene 1 group (B1: 150 mg/kg b.w.) and Benzene 2 group (B2: 300 mg/kg b.w.) [26,27]. Mice were injected subcutaneously with either corn oil or a benzene-corn oil mixture once daily, 5 days per week for 3 months. The mice were observed for the following 10 months after exposure to benzene. The body weight was recorded every month. All procedures were approved by the animal care and use committees of the Southeast University (Approval No: 20130027).

### 2.3. Blood Parameters, Relative Organ Weight and Bone Marrow Smears

The mice were anesthetized with pelltobarbitalum natricum, and blood was collected into an anticoagulation tube for blood test (WBCs, RBCs, platelets (Pit) and hemoglobin (Hgb) level). Then the mice were sacrificed by decapitation. Liver, spleen, lung, and kidney were excised and weighed. Relative organ weight was calculated as the ratio between organ weight and body weight. Bone marrow cells were flushed from one tibia using a 23-gauge needle to make smears. The bone marrow smears were stained with Wright-Giemsa and used for determination of differential blood counts.

### 2.4. Collection of Hematopoietic Stem cells

Bone marrow (BM) cells were harvested from mice femur and tibia, flushed with stain buffer (fetal bovine serum (FBS)) (BD Pharmingen, San Jose, CA, USA), and pipetted gently. The70 μm cell strainer (BD Pharmingen, San Jose, CA, USA) was used to get a single cell suspension. To get the hematopoietic stem and multipotent cells (LSK cells), the BM cells were then stained with fluorescent-labeled antibody namely APC lineage cocktail, Sca-1 PE-CY7 and c-Kit PE-CY5 (BD Pharmingen, San Jose, CA, USA) and incubated at 4 °C in the dark for 45 min. The cells were washed twice and resuspended in 1 mL stain buffer. Then one drop of 7-AAD was added for 5 min. BD FACS Aria Ⅱ Cell Sorter (BD Bioscience, San Jose, CA, USA) were used for flow cytometric analysis and cell sorting. The purity of isolated LSKs that was routinely obtained was >97%.

### 2.5. Colony Forming Cells Assay

A total of 2.5 × 10^4^ BM cells were plated in triplicate in 35-mm non tissue culture plates in methylcellulose medium (M3434) supplemented with recombinant cytokines according to the manufacturer’s instructions (Stem Cell Technologies, Vancouver, BC, Canada). Myeloid and erythroid colonies formation was scored after 12 days of culture.

### 2.6. Real-time PCR Assay

Total RNA of LSK cells (10^4^ cells for each sample) was isolated using RNA Isolation Kit (Ambion, Austin, TX, USA), then reverse-transcripted with High Capacity cDNA Reverse Transcription Kit (Applied Bio-systems, Carlsbad, CA, USA). A two step, nested real-time PCR was performed using SYBR-Green Real-time Master Mix (Toyobo, Japan). A standard nested PCR protocol was followed. External and internal primers were designed using Primer 5 software (see Table 1). For the second round real-time PCR assay, the reactions were performed on an ABI 7500 system. The cycling conditions were as follows: 30 s at 95 °C for pre-denaturation,30 cycles for 15 s at 95 °C for denaturation,1 min at 60 °C for annealing and 10 s at 72 °C for elongation. The fluorescence emitted by the SYBR Green was measured at the end of each cycle. The relative gene expression was analyzed by the 2^−^^△△^^Ct^ method.

### 2.7. Statistical Analysis

All the values are presented as mean ± standard deviation. Results were analyzed by one-way Anova except as noted. Statistical analysis was performed using SPSS 16.0 software (SPSS, Chicago, IL, USA). A value of *p* < 0.05 was considered to be statistically significant.

**Table 1:** All outer (or nesting) primer pairs and the Real time-PCR primers used in this study.

Primer Name	Primer Set	Sequence (5′-3′)
β-actin	Outer	Forward: CGAGCGGTTCCGATGCCCTG; Reverse: ACGCAGCTCAGTAACAGTCCGC
	Inner	Forward: ATCCGCAAAGACCTGT; Reverse: GGGTGTAACGCAACTAAG
β-catenin	Outer	Forward: GACACCTCCCAAGTCCTTT; Reverse: CCTCTGAGCCCTAGTCATT
	Inner	Forward: ACACCTCCCAAGTCCTTTATGAAT; Reverse: CCCGTCAATATCAGCTACTTGCT
c-Myc	Outer	Forward: GCGCTCTCCTTCCTCGGACTCGC; Reverse: GGTTTGCCTCTTCTCCACAG;
	Inner	Forward: GCCCCTAGTGCTGCATGAG; Reverse: CCACAGACACCACATCAATTTCTT
Notch1	Outer	Forward: GGCCTTGAGACAGGCAACA; Reverse: CTGCACTGGCCTCCAGCAA
	Inner	Forward: CCTTGAGACAGGCAACAGTGAAG; Reverse: GAGAGTATCGGGCAGCCAAGT
cyclinD1	Outer	Forward: GCGCTTCCAACCCACCCTCCATG; Reverse: GCGCCGCAGGCTTGACTCCAGAA
	Inner	Forward: GGGCAGCCCCAACAACTT; Reverse: GCAGTCCGGGTC ACACTTG
Bmi1	Outer	Forward: CAGAGGGATGGACTGACGA; Reverse: GCGTGAGGGAACTGTGGGTGA
	Inner	Forward: GAAAGTGACTCTGGGAGTGACAAG; Reverse: GGGACTGGGCAAACAGGAA
p53	Outer	Forward: CTTCCACCTGGGCTTCCTG; Reverse: GTGGCGCTGACCCACAACT
	Inner	Forward: TGGCGCTGACCCACAACT; Reverse: CCAAGTCTGTTATGTGCACGTACTCT

## 3. Results

### 3.1. Body Weight and Relative Organ Weight

**Figure 1:**
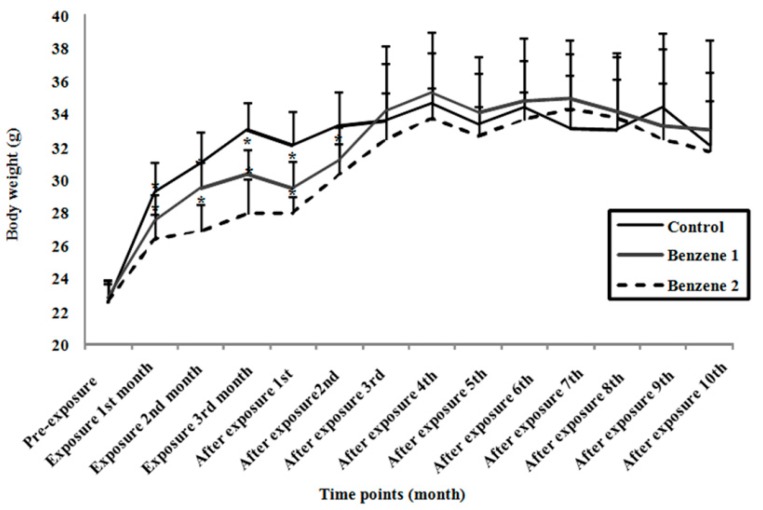
Body weight of C3H/He mice at different time points. The body weight was monitored each month. Values are expressed as mean ± standard deviation (SD). Control: *n* = 14; Benzene 1: *n* = 12; Benzene 2: *n* = 9 (* *p* < 0.05 compared with control.).

Mice in Benzene groups showed varying degrees of irritability and lethargy. The body weight was decreased by about 8% at 150 mg/kg/day and about 15% at 300 mg/kg/day at the end of benzene exposure (Figure 1). Then no significant difference was observed at 10-months post benzene exposure. The relative organ weights were presented in Table 2 and as shown, only mice in Benzene 2 group showed statistically significant increase in relative spleen weights at 10-months post benzene exposure.

**Table 2:** Relative organ weight of mice at 10 months post-benzene exposure.

Group	Relative Organ Weight (Mean ± SD)			
	Relative Liver Weight	Relative Spleen Weight	Relative Lung Weight	Relative Kidney Weight
Control	5.31 ± 0.89	0.38 ± 0.10	0.88 ± 0.44	1.93 ± 0.10
Benzene 1	5.14 ± 0.39	0.41 ± 0.10	0.91 ± 0.44	1.92 ± 0.17
Benzene 2	5.37 ± 0.57	0.52 ± 0.14*^,#^	0.98 ± 0.43	1.89 ± 0.25

Relative organ weight = (absolute organ weight × 100%) / body weight of mice on the day of sacrifice. Control: *n* = 14; Benzene 1: *n* = 12; Benzene 2: *n* = 9. * *p* < 0.05 compared with control; ^#^ *p* < 0.05 compared with B1 group.

### 3.2. Blood Parameters and Bone Marrow Smears

After exposure to benzene for 3 months, a significant decline in WBC, RBC, Pit counts and Hgb level were observed in benzene groups. However, at 10 months post-benzene exposure, the WBC counts in benzene groups together with the RBC counts and Hgb level in Benzene 1 group returned to normal range. Meanwhile, the RBC counts and Hgb level were remained slightly lower in Benzene 2 group. As for platelet count, a dose-dependently increment following benzene exposure was observed (Table 3).

**Figure 2:**
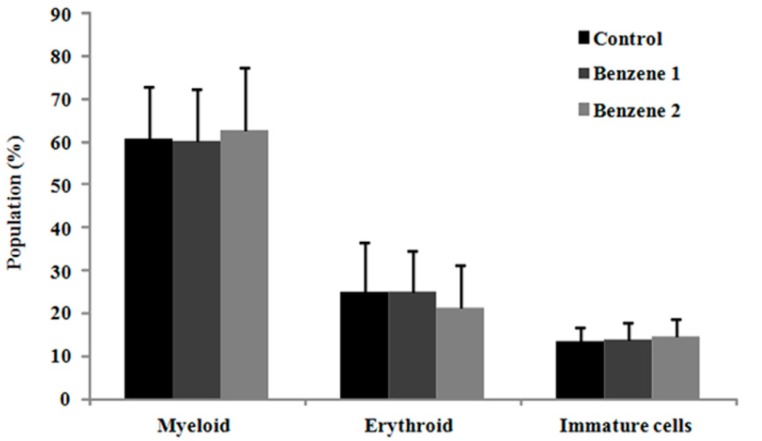
Bone marrow smear examination of mice at 10 months post-benzene exposure.

In previous studies [28,29], we found benzene exposure could cause various level of erythroid or myeloid hyperplasia. In present study, no significant difference was observed in the frequency of myeloid, erythroid and immature cells between control and benzene groups at 10 months post benzene exposure (Figure 2).

### 3.3. Frequency of LSK Cells and Number of Colony Forming

**Figure 3:**
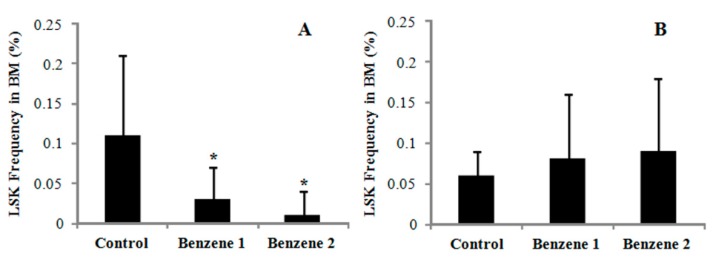
Frequency of LSK cells in mice BM. (**A**) after benzene exposure for 3 months; (**B**) after 10 months post-benzene exposure. Control: *n* = 14; Benzene 1: *n* = 12; Benzene 2: *n* = 9.* *p* < 0.05 compared with control.

**Figure 4:**
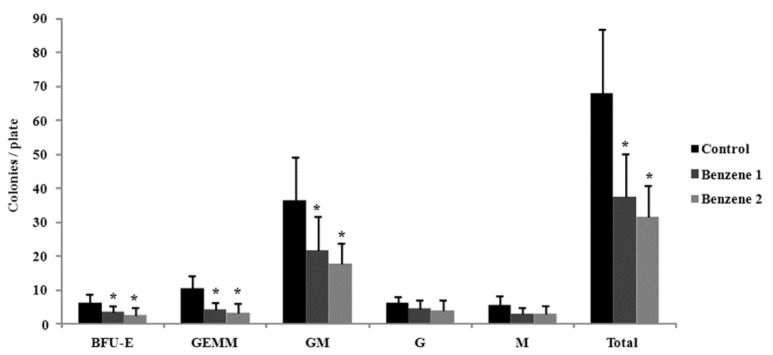
Colony-forming capability of mice after 10 months post-benzene exposure. * *p* < 0.05 compared with control.

The frequency of LSK cells was dramatically decreased in Benzene groups after 3 months of benzene exposure. While at 10 months post benzene exposure, the frequency of LSK cells in the BM was slightly increased in Benzene groups although no statistical difference was noted as compared to Control group (Figure 3). To assess progenitor function, we performed colony forming cells assay, enumerating both erythroid burst-forming unit (BFU-E) as well as the committed progenitors granulocytes-erythroid-monocyte-megakaryocyte (GEMM), granulocyte-macrophages (GM), granulocytes (G), and macrophages (M) (Figure 4). By 10 months post-benzene exposure, the BM cells were harvested and plated in cytokine-supplemented methylcellulose. Twelve days later, the G and M progenitors were decreased but no significant differences compared with control, whereas BFU-E, CFU-GEMM and CFU-GM progenitors showed significantly dose-dependent decline in the benzene groups. Although no significant changes were found in percentage of HSC cells, the colony formation ability of progenitors was dramatically declined even after all benzene exposure has been stopped for ten months.

**Table 3:** Blood parameters of mice after benzene exposure for 3 months and at 10 months post-benzene exposure.

Time Points	Blood Parameters (Mean ± SD)				
	Group	WBC (10^9^/L)	RBC (10^12^/L)	Hgb (g/L)	Pit (10^9^/L)
Three months after benzene exposure	Control	5.05 ± 1.56	8.28 ± 0.32	138.42 ± 4.07	661.92 ± 87.01
	Benzene 1	1.60 ± 0.72*	5.63 ± 1.15*	114.00 ± 19.8*	374.00 ± 144.18*
	Benzene 2	2.19 ± 1.16*	5.84 ± 1.39*	90.00 ± 37.98*	318.67 ± 163.54*
Ten months post-benzene exposure	Control	7.43 ± 2.03	8.23 ± 1.22	130.27 ± 22.12	514.40 ± 85.60
	Benzene 1	5.81 ± 1.82	7.59 ± 1.07	117.75 ± 16.12	677.37 ± 155.22*
	Benzene 2	6.10 ± 2.49	6.75 ± 1.27*^,#^	106.65 ± 13.10*	701.73 ± 196.21*

Control: *n* = 14; Benzene 1: *n* = 12; Benzene 2: *n* = 9. * *p* < 0.05 compared with control; ^#^ *p* < 0.05 compared with B1 group.

### 3.4. Expression of β-catenin, c-Myc, Notch1, Cyclin D1, Bmi 1 and P53

**Figure 5:**
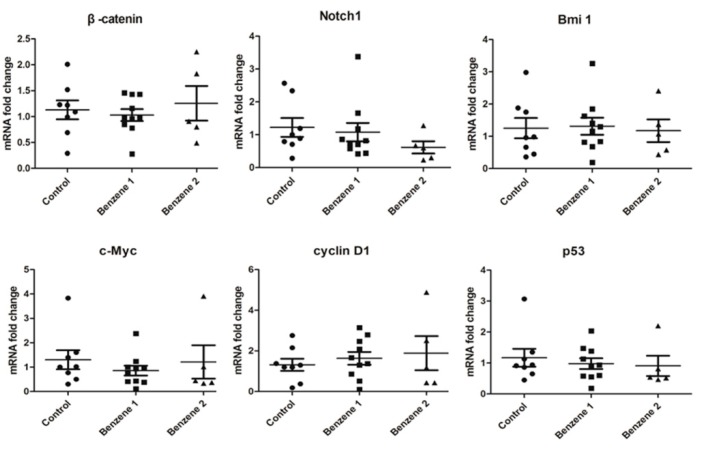
β-catenin, c-Myc, Notch1, cyclin D1, Bmi 1 and p53 gene expression in mouse LSK cells following 10 months post-benzene exposure.

The nest real-time PCR was used to detect expression of 6 genes in Wnt/β-catenin, Notch and Hh signaling pathways. In this study, the mRNA level of β-catenin in LSKs was found declined slightly in B1 group and elevated in B2 group without significant differences (Figure 5).The specific known target genes of Wnt signaling include c-myc and cyclin D1. The expression of c-myc was decreased only in B1 group and no significant difference was found in B2 group. Elevated levels of cyclin D1 were detected in benzene groups with benzene dose increased, but the difference was not statistically significant because of variability between mice. Our results showed that the mRNA level of Notch1 was decreased in mice exposed to benzene. For the Bmi 1 mRNA expression, no statistical difference was found between these three groups. The mRNA level of p53, which is one of the target genes of Bmi 1, was lower than that of the non-exposure controls.

## 4. Discussion

Benzene exposure may perturb biological processes, gene expressions and signaling pathways which may lead to hematotoxicity, leukemia and lymphoma. When individuals are exposed to benzene, repair mechanisms function by means of antioxidation, DNA-repair and autophagy will be activated [8,30,31]. Excessive exposure will result in increased damage to cells aggravated by a defective repair mechanism. Benzene induced alterations in target genes or pathways in HSCs may change the normal hematopoiesis and lead to exhaustion of HSCs with normal self-renewal and differentiation ability under stress condition [32,33]. It is noteworthy that hematopoietic diseases may occur years later even after stopping the benzene exposure for long term [32]. Therefore, the accumulation of damage in HSCs with age resulting in persist hematotoxicity may play a central role in the initiation of benzene-induced leukemia.

In the present study, the blood counts analyses were conducted at 3 months after benzene exposure and after 10 months post-benzene exposure. After benzene exposure for 3 months, statistically significant decreases in WBC, RBC, Pit counts and Hgb level were observed. By 10 months post benzene, the WBC level in Benzene groups recovered to normal level. While the RBC and Hgb levels in Benzene 2 group remained lower by 10 months after stopping benzene exposure. Pit numbers tended to increase by 10 months post-benzene exposure in Benzene groups. Compared with leukocytes (WBC), stronger benzene effects for erythrocytes (RBC and the level of Hgb) were found. The selective effects which are cell-type lineage dependent are consistent with several previous literatures [34,35].

Hematopoiesis occurs in the bone marrow. Precursor cells undergo proliferation to ensure the proper number and type of circulating blood cells can be produced. Compared with blood parameters, a smear of bone marrow contains many precursor cells at late stages of differentiation, with few HSCs and cells at early stages of differentiation. After benzene exposure for 30 days, a significant erythroid hyperplasia in Benzene 2 group and higher percentage of immature cells in Benzene groups were observed [28]. While the results of bone marrow smears by 10 months post exposure showed no difference in the percentage of myeloid, erythroid and immature cells in benzene groups. This may indicate that the cells in late stage of differentiation and peripheral blood gradually recover to normal level after 10 months from the last benzene exposure. It was still unknown if the toxic effects of benzene on HS/PCs exist or not. So we analyzed the percentage of LKS cells, performed the colony-forming assay and detected the expression of several key genes in proliferation signaling pathways using nest real-time PCR.

HSCs arise in the bone marrow and were long thought as a homogeneous population of cells [32]. Since benzene can cause a decrease in the three major circulating cells types, it may affect hematopoietic stem and progenitor cells, the populations which are considered to be candidates for the accumulation of multistep, genetic mutations [32,33]. The proportion of LSK cells were statistically lower in Benzene groups at 3 months after benzene exposure and dose-dependently increased following 10 months post-benzene exposure, although the latter was not statistically significant. This increase in the frequency LSK during the post exposure stage may be due to proliferative expansion of HSCs under stress condition.

Colony forming units (CFUs) are an indicator of stem cells’ capacity for both self-renewal and differentiation. Martyn’s group observed highly significant, dose-dependent decreases in progenitor cell colony formation in workers exposed to benzene [36]. In our case, statistically significant decreases in BFU-E, CFU-GM and CFU-GEMM were also observed even after stopping benzene exposure for 10 months, while the CFU-G and CFU-M were not remarkably affected. As the progenitors that produce CFU-GM and CFU-GEMM are more immature cells than those produce CFU-G and CFU-M, this indicates the inhibition effects of benzene on colony capacity of early stage progenitors tended to last longer than those late stage progenitors. Benzene-induced ROS formation may be one of the reasons for decreased colony forming ability. Helen *et al.* found the abnormal colony growth was abrogated by pretreatment with PEG-catalase (antioxidative anzyme) [37]. The other factors which contribute to this need to be further studied. Compared with blood parameters (Table 2), a greater proportional decrease was observed in colony formation than in levels of mature cells in peripheral blood, indicating that early progenitor cells are more sensitive than mature cells to the hematotoxic effects of benzene. Furthermore, although a portion of LSK cells recovered after 10 months off exposure, delayed effects of benzene on function of progenitors persist.

The Wnt/β-catenin, Notch and Hh signaling pathways play roles in self-renewal and differentiation in HSCs. Thus expression changes in key and target genes in these three pathways may contribute to the development of benzene-induced leukemia. The Wnt/β-catenin pathway is active in normal HSCs and has previously been implicated in the development of human leukemia [10,11]. Jamieson *et al.* reported the aberrant activation of Wnt/*β*-catenin pathway may result in enhanced self-renewal capability in GM progenitors in CML [38]. Maria *et al.* found varying levels of β-catenin protein in primary AML blasts, and the β-catenin pathway may be involved in AML pathogenesis [39]. Wang and colleagues suggested that reactivation of β-catenin signaling was required for the transformation of progenitor cells by certain oncogenes [40]. In the present study, the expression of *β*-catenin in Benzene 2 group increased but without significant difference compared with control. Next, we assessed the expression of c-Myc and cyclin D1, two target genes of Wnt/β-catenin pathway. A slight drop in c-Myc expression was only found in Benzene 1 group, and the expression of cyclin D1 in LSK cells increased in a dose-related manner in Benzene groups. Endogenous c-Myc is differentially expressed and induced upon differentiation of long-term HSCs. Anne *et al.* found conditional elimination of c-Myc activity in the BM resulted in cytopenia and accumulation of HSCs, possibly due to their failure to initiate normal stem cell differentiation [41]. Levels of expression of c-Myc in LSK cells are different between two Benzene groups, which may suggest benzene dosage has a direct effect on gene expression and disease progression.

As one of the downstream genes in the Notch pathway, cyclin D1 promote progression to the G1 phase of the cell cycle on the basis of its cyclic pattern of mRNA expression. Cyclin D1 is also considered to be a key oncogene, whose function links the cell cycle to proliferation, apoptosis and differentiation [42,43]. Upregulation of cyclin D1 has been shown to induce a rapid cell cycle and increase cell proliferation rate. Saha *et al.* found higher protein level of cyclin D1in benzene-induced mice compared with control mice [44], which is consistent with our observation. Over-expressed of cyclin D1 was also found in high risk MDS and trisomy 8 AML [19,20]. Up-regulation of cyclin D1 increases proliferation of leukemia and lymphoma cells, and may upregulate anti-apoptotic proteins, leading to a block in apoptosis. The elevated cyclin D1 in our study may indicate deregulation of cell cycle and apoptosis in HS/PCs, which may be responsible for the pathogenesis of benzene-induced leukemia. Notch1 is crucial regulator in T-cell differentiation and proliferation, dysregulation of Notch signaling has been reported in haematologic malignancies. Chen and colleagues found down-regulation of Notch1 expression may be a key molecular event in the leukemogenesis of AML [45]. Moreover, Lobry *et al.* showed that Notch signaling was silenced in human AML samples as well as in AML-initiating cells in animal model [18]. In the present study, we found that the down-regulation of Notch1 in Benzene groups which may be related to aberrant self-renew and differentiation of HSCs.

Hh pathway is required for self-renewing of HSCs in order to modulate cell cycle regulators. Deregulated Hh signaling has been related to transformation and tumor maintenance in several cancers [23]. As one of major downstream effectors of Hh, Bmi 1 was reported to be enhanced during the progression of CML through transcriptional and posttranscriptional regulation [46]. Saudy *et al*. detected higher expression of Bmi 1 in AML and CML patients and concluded that detection is helpful for predicting the survival in AML patients and monitoring the aggressiveness and progression in CML patients [47]. In our case, no significant changes in Bmi 1 were detected in benzene exposure groups, this may be explained by the different extent of hematopoietic damage and early stage progression of hematopoietic disorders caused by benzene.

The tumor suppressor p53 is a target gene of Bmi 1. p53 is a key transcription factor that plays an important role in regulating HSC quiescence and self-renewal during normal (steady-state) hematopoiesis [24]. In addition, p53 triggers cells protective actions including cell-cycle arrest, DNA repair, or induction of apoptosis under stress conditions. p53 was reported to decrease ROS levels, thereby protecting HSCs from DNA damage [48]. Loss of p53 is related to decreased apoptosis and increased disease progression of leukemic cells [24,49]. Furthermore, p53 loss was reported to promote HSC proliferation, impair HSC functions with an increase in hematologic tumors [50]. Our results showed that the mRNA expression of p53 in benzene exposed group declined. Accumulation of ROS is involved in the benzene induced toxicity [33]. The reduction in p53 mRNA level may result in failed entry into apoptosis and protect LSK cells from DNA damage, thus leading to impairment of LSK functions.

Although the expression changes in these 6 genes in LSK cells of mice post benzene exposure are not as significant as they do in leukemia patients, these changes may be one of the key events occurring in the progression of benzene-induced hematological disorders and can be potential indicators for pre-clinical disease progression.

## 5. Conclusions

In this study, we focused on investigating the potential toxic effects of benzene on colony-forming ability, proliferation-associated gene expression in HS/PCs in mice following 10 months post-benzene exposure. The BFU-E, CFU-GEMM and CFU-GM progenitors were significantly dose-dependent decreased, while the CFU-G and CFU-M progenitors were not remarkably affected. The cyclin D1 was increased, the Notch1 and p53 mRNA level in LSK cells were decreased, although no statistical difference was found. The alterations in these genes may affect apoptosis, cell cycle, proliferation and differentiation in HS/PCs, allow pre-leukemic clones to escape elimination and finally lead to increased risk of neoplastic transformation. Our findings suggest the hematotoxic effects on LSK cells induced by chronic benzene exposure persist even long term after the exposure ceases.
